# Complete transection of the bilateral main bronchus in a 5-year-old patient: a case report

**DOI:** 10.1186/s13019-024-02828-2

**Published:** 2024-06-25

**Authors:** Weimin Wang, Yanliang Yang, Siming Bi, Xiaozheng Lv, Huihui Xu

**Affiliations:** Department of Cardiac Surgery, Children’s Hospital Affiliated Shandong University, (Jinan Children?s Hospital), No. 23976, Jingshi Road, Huaiyin District, Jinan City, Shandong Province China

**Keywords:** Thoracic trauma, Electronic fibreoptic bronchoscopy, Tracheobronchial injury, Tracheoplasty, Extracorporeal circulation

## Abstract

**Background:**

Tracheobronchial injuries caused by blunt chest trauma are rare in children, and such injuries usually involve multiple organs. Most cases involve respiratory failure on the way to the hospital, and the mortality rate is high. Herein, we describe the case of a 5-year-old patient who fell from an electric vehicle, causing complete rupture of the bilateral main bronchus.

**Case presentation:**

We treated a 5-year-old patient with complete bilateral main bronchus rupture. Chest computed tomography (CT) failed to detect bronchial rupture. Continuous closed thoracic drainage resulted in a large amount of bubble overflow. Tracheal rupture was suspected. Fibreoptic bronchoscopy revealed complete rupture of the right main bronchus and rupture of the left main bronchus. Emergency tracheoplasty was performed under cardiopulmonary bypass (CPB). During the operation, we found that the bilateral main bronchi were completely ruptured. Postoperative recovery was smooth. The traditional surgical method for treating these injuries is lateral thoracotomy. However, a median sternotomy provides a better opportunity for selective repair. Extracorporeal circulation-assisted surgery is required for patients with unstable breathing.

**Conclusion:**

Complete fractures of the bilateral main bronchi are rare. Bronchial rupture should be suspected in the presence of expansion defect-dropped lungs and massive air leakage despite tube thoracostomy in haemopneumothorax developing after thoracic trauma. Extracorporeal circulation-assisted tracheoplasty is a relatively safe option for children whose respiratory system is difficult to maintain, thus ensuring oxygenation ventilation and a clear surgical field.

**Supplementary Information:**

The online version contains supplementary material available at 10.1186/s13019-024-02828-2.

## Introduction

Traumatic bronchial injury is a serious complication of blunt chest trauma, and this injury is rare among children. The injury often leads to severe respiratory distress and may even be life threatening. The primary management of these injured patients is to rebuild airways to maximize the retention of a functioning lung [1]. In cases of unstable breathing, the use of extracorporeal circulation increases safety during the operation and encourages repair rather than resection of the affected lung [2]. The purpose of this study was to introduce our treatment and surgical experience in the treatment of a child with complete rupture of the bilateral main bronchus.

### Case presentation

We treated a 5-year-old patient who was transferred to a surrounding hospital. He had fallen from a three-wheeled car and gradually developed dyspnoea and wheezing on the way to the local hospital. Closed thoracic drainage, tracheal intubation, and mechanical ventilation were performed at the local hospital. The child has been in good physical health in the past. After 3 days of poor treatment response, the patient was transferred to the critical care unit of our hospital.

**Fig. 1:**
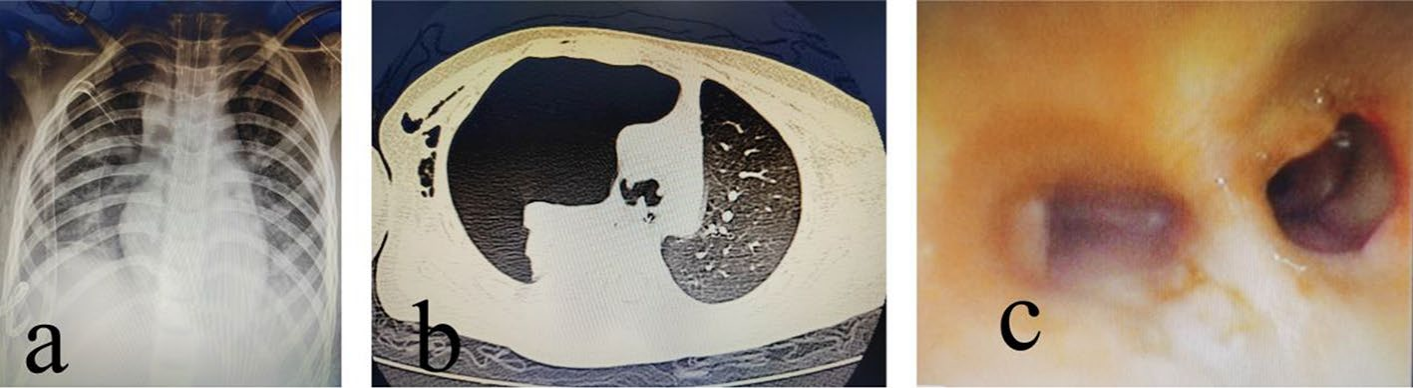
(**a**) Preoperative X-rays showed two drainage tubes inserted after treatment for right pneumothorax (**b**) Chest CT showed right pneumothorax (**c**) Bronchoscopic image of main bronchus rupture

Bedside chest radiography revealed two drainage tubes inserted after treatment for right pneumothorax (Fig. 1a). Chest computed tomography (CT) revealed a right pneumothorax with compression atelectasis (Fig. 1b). The drainage tube continued to leak, and tracheal injury was suspected. Electronic bronchoscopy revealed complete rupture of the right main bronchus near the carina from the opening and visible rupture of the left main bronchus from the opening (Fig. 1c). We performed emergency surgery. No obvious anticoagulation contraindications were found after multidisciplinary consultation.

**Fig. 2:**
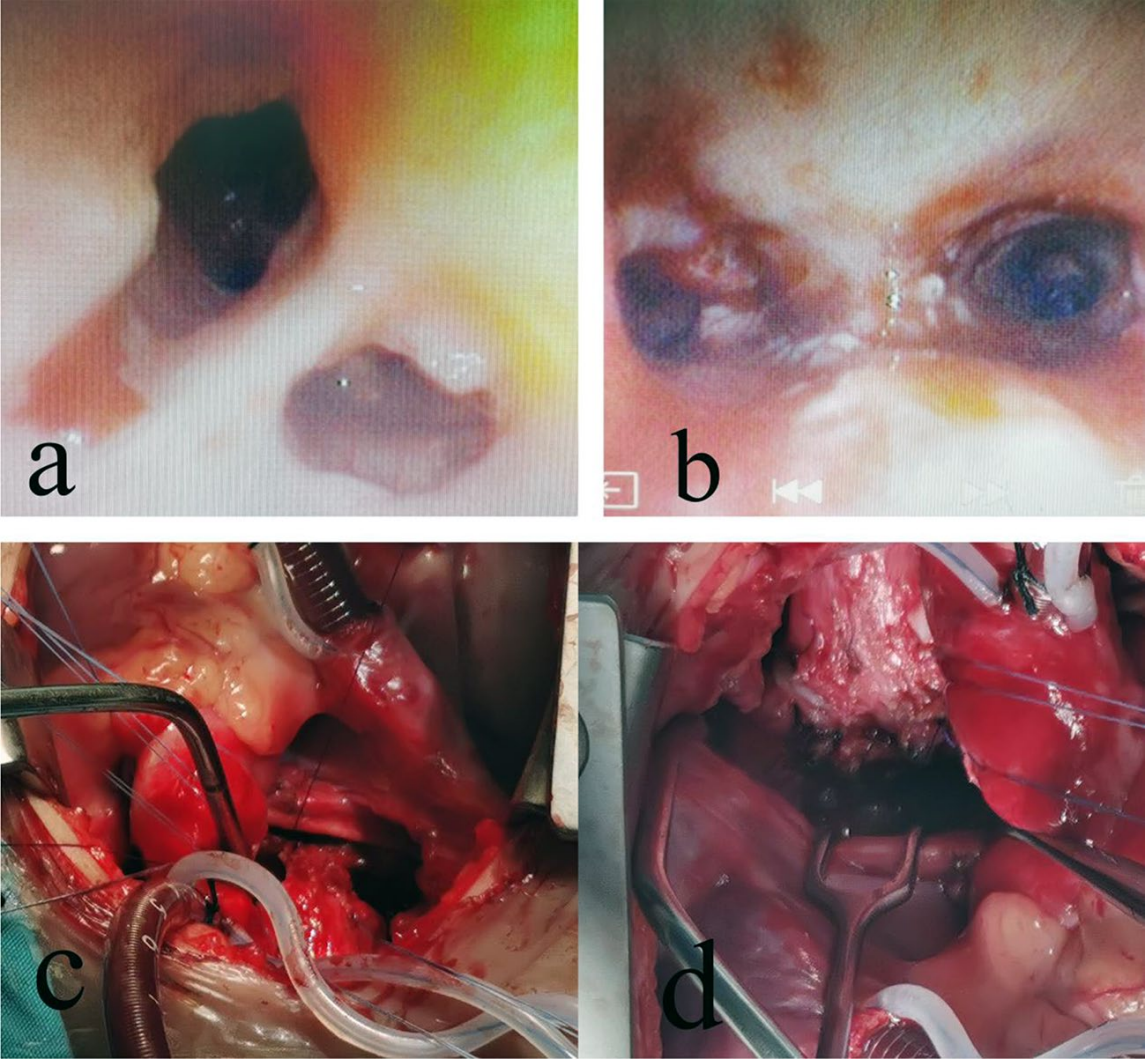
(**a**) Intraoperative bronchoscope image of main bronchus rupture (**b**) Postoperative bronchoscope image of main bronchus after suture (**c**) Intraoperative view of main bronchus rupture before suture (**d**) Intraoperative view of main bronchus rupture after suture

Double-lumen intubation was unsuccessful during surgery. Considering the child’s severe condition, the rupture of the bilateral main bronchi, the long period after the trauma, and the difficulty of anastomosis during the operation, further cardiopulmonary deterioration was expected. We decided to open the chest in the middle of the emergency period and prepare for cardiopulmonary bypass. During the operation, a large amount of air leaked into the trachea after the free part of the carina, and the ventilator could not maintain a sufficient tidal volume. After the wet gauze was pressed, we established a cardiopulmonary bypass. Cannulation of the vessels was made in auricula dextra and aorta, the blood volume was insufficient, and cannulation of the vessels was increased in inferior vena cava. During surgery, we confirmed that the left and right main bronchi were completely disrupted from the opening. The distances between the distal and proximal ends were approximately 2.5 cm and 1.6 cm, respectively. A large amount of old bloody and purulent secretions was observed in the lumen (Fig. 2a, b). The 4-PDS absorbable suture was used for continuous anastomosis of the posterior wall of the left and right main bronchus, and the anterior wall was intermittently sutured(Fig. 2c, d).

**Fig. 3:**
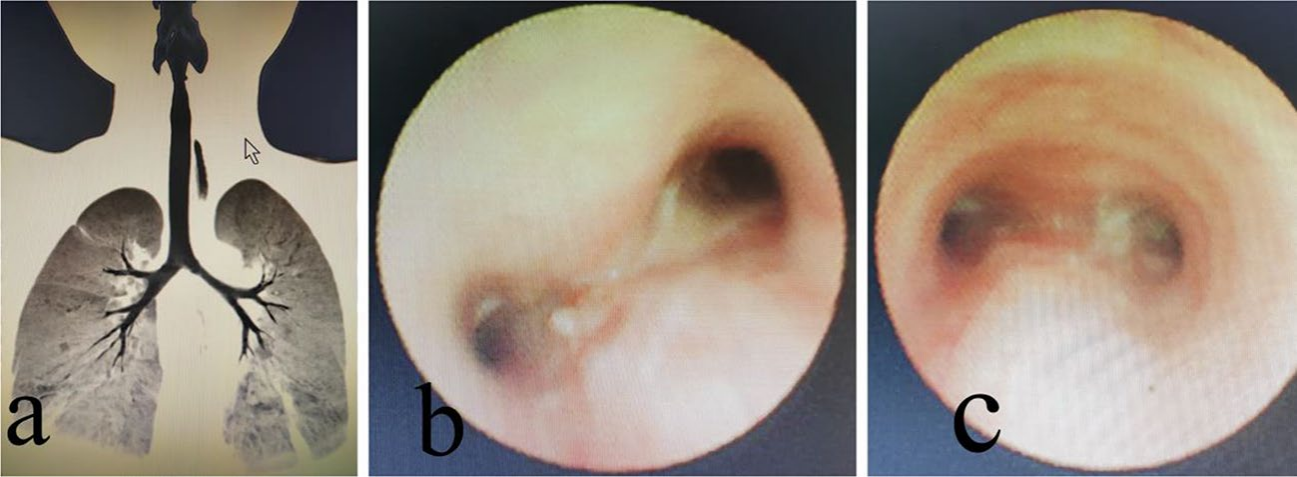
(**a**) A postoperative coronal CT scan (**b**) A bronchoscopy image 2 months after surgery (**c**) A bronchoscopy image 3 months after surgery

On the first day after surgery, an invasive ventilator was withdrawn for mechanical ventilation. After weaning, breathing was slightly laborious. Noninvasive ventilation was administered for 6 days. An early chest radiograph revealed pneumonia, which disappeared after intravenous antibiotics and chest physical therapy. Chest CT revealed complete expansion of the lungs (Fig. 3a). The patient was discharged 21 days after treatment. Electronic bronchoscopy performed 2 months after the operation revealed an unobstructed bilateral main bronchial lumen (Fig. 3b). More than 3 months after the operation, electronic bronchoscopy revealed an unobstructed bilateral main bronchial lumen and a small scar (Fig. 3c). We followed this patient for 3 months. The child had stable breathing and physical strength similar to those of healthy children of the same age.

### Discussion and conclusion

Tracheobronchial disruption (TBD) accounts for 0.5-2.8% of blunt chest traumas [3]. The mechanism of TBD involves bulging traction, accelerated deceleration resulting in shear stress, or a sudden increase in intrathoracic pressure. These forces can work alone or in combination [4]. In blunt trauma, 75-90% of TBDs are located within 2–3 cm of the carina, and 60% are located within 1 cm [5].

Symptoms depend on whether there is communication between the injured airway and the pleural cavity [6]. Subcutaneous emphysema and dyspnoea are the most common initial signs of bronchial injury. Massive air leakage or persistent atelectasis may cause severe airway injury [7]. Our patient developed dyspnoea and massive air leakage in the chest tube after trauma, and tracheal injury was suspected.

Chest CT is superior to chest X-ray and can identify lesions and detect concomitant injuries. The sensitivity of CT imaging is 71-100% [8]. However, despite bronchial injury, the connective tissue around the bronchus may remain intact and allow distal lung ventilation [6]. Due to surrounding tissue oedema and bleeding, there are still some false-negative results, and CT-negative results do not rule out the presence of tracheal injury. In this case, chest CT showed right hydropneumothorax with right lung compression atelectasis but failed to show bilateral main bronchus rupture. There was no gas leakage on the left side due to the surrounding tissue encapsulation, and no imaging signs such as pneumothorax or atelectasis were observed.

Electronic fibreoptic bronchoscopy is a relatively accurate tool for diagnosing tracheobronchial injuries. Patients with persistent thoracic air leakage should undergo bronchoscopy immediately [9]. Electronic bronchoscopy revealed complete rupture of the right main bronchus from the carina and visible rupture of the left main bronchus in the carina. We confirmed that the left and right main bronchi had ruptured completely from the protuberance. The distances between the distal and proximal ends were approximately 2.5 cm and 1.6 cm, respectively. As bronchoscopy depends on personal experience, errors may occur.

A delayed diagnosis is defined as a definite diagnosis more than 48 h after injury and is common in patients transferred from surrounding hospitals [10]; 25-68% of patients are not diagnosed with tracheal injury within this time [11]. A delay in diagnosis results in scar tissue and obstruction of the bronchus by granulation tissue [12]. Therefore, timely diagnosis and treatment are essential to avoid scarring or granulation tissue hyperplasia, ensure the repair of damaged bronchi, and maximize the retention of lung tissue. This child was transferred from another hospital, which contributed to delayed diagnosis; he was diagnosed on the 6th day after trauma. This greatly increased the probability of scarring or granulation tissue blocking the airway, which was a key factor in our decision to perform extracorporeal circulation-assisted surgery.

Patients with tracheal injuries have been reported to undergo repair under cardiopulmonary bypass [2]. Considering the following points, we chose extracorporeal circulation surgery: (1) If the trachea is completely transverse, the distal end may slip into the mediastinum [8]. Our intraoperative electronic bronchoscopy results are consistent with these findings. Disruptions at the bifurcation site may require a double-lumen tube or selective endobronchial intubation [13]. Multiple attempts at double-lumen intubation were unsuccessful. The distal trachea may slide into the mediastinum, and selective endobronchial intubation is difficult; this may lead to secondary injury [14]. (2) The best time for surgery is within 48 h, allowing good repair and retention of lung tissue [15]. The child was diagnosed 6 days after the trauma, increasing the incidence of complications such as scarring and stenosis. Extracorporeal circulation surgery can guarantee good vision, provide good ventilation, ensure suture-free accuracy (as much as possible) to repair damage rather than remove lung tissue lesions, and reduce postoperative complications. (3) There was a large amount of air leakage after intraoperative free bulge. It is difficult for ventilators to maintain tidal volume to avoid rapid cardiopulmonary deterioration and hypoxia, leading to severe cardiac arrest, etc. This life-threatening situation can be avoided only through the emergency establishment of cardiopulmonary bypass to restore adequate oxygenation.

From our experience in dealing with congenital tracheal stenosis, we have summarized the following points. (1) The anterior wall of the broken end was sutured intermittently. Posterior wall anastomosis was difficult. We chose continuous suturing of the posterior wall, which reduces the difficulty of suturing, ensures the smoothness of the posterior wall, and reduces anastomotic dehiscence and granulation tissue hyperplasia. (2) Excessive airway dissociation should be avoided to prevent vascular stripping, which may result in insufficient blood supply, anastomotic dehiscence, or nonunion. (3) The patient should be fully free under the condition of ensuring that the blood supply to avoid anastomotic tension is too high, resulting in anastomotic dehiscence. (4) Absorbable sutures and extratracheal knotting were used during surgery to avoid granulation tissue hyperplasia caused by nonabsorbable sutures.

Complications, including early anastomotic dehiscence or airway stenosis, develop in up to 25.8% of patients [5]. The incidence of stenosis and anastomotic dehiscence is 5-6% [16]. Although diagnosis was delayed in our patient, intraoperative cardiopulmonary bypass was successful, and the sutures were exact and meticulous, preventing complications. Postoperative chest CT and electronic bronchoscopy revealed satisfactory tracheal morphology.

In summary, patients with complete fractures of the bilateral main bronchi are rare. A median sternotomy provides a better opportunity for selective repair. Extracorporeal circulation-assisted tracheoplasty is necessary for the treatment of bilateral main bronchial injuries involving an unstable respiratory status. We ensured adequate tracheal blood supply and complete dissociation during surgery. Intermittent sutures combined with continuous sutures were used to avoid complications such as anastomotic dehiscence and granulation.

### Electronic supplementary material

Below is the link to the electronic supplementary material.

Supplementary Material 1

Supplementary Material 2

Supplementary Material 3

Supplementary Material 4

## Data Availability

The datasets used and/or analysed during the current study are available from the corresponding author upon request.

## Abbreviations

CT: Chest computed tomography
CPB: cardiopulmonary bypass
TBD: tracheobronchial disruption
