# Supplementary figures and images for: Complete transection of the bilateral main bronchus in a 5-year-old patient: a case report
Source: J Cardiothorac Surg. 2024 Jun 25;19:373. doi: 10.1186/s13019-024-02828-2 (PMC11197318; doi:10.1186/s13019-024-02828-2)

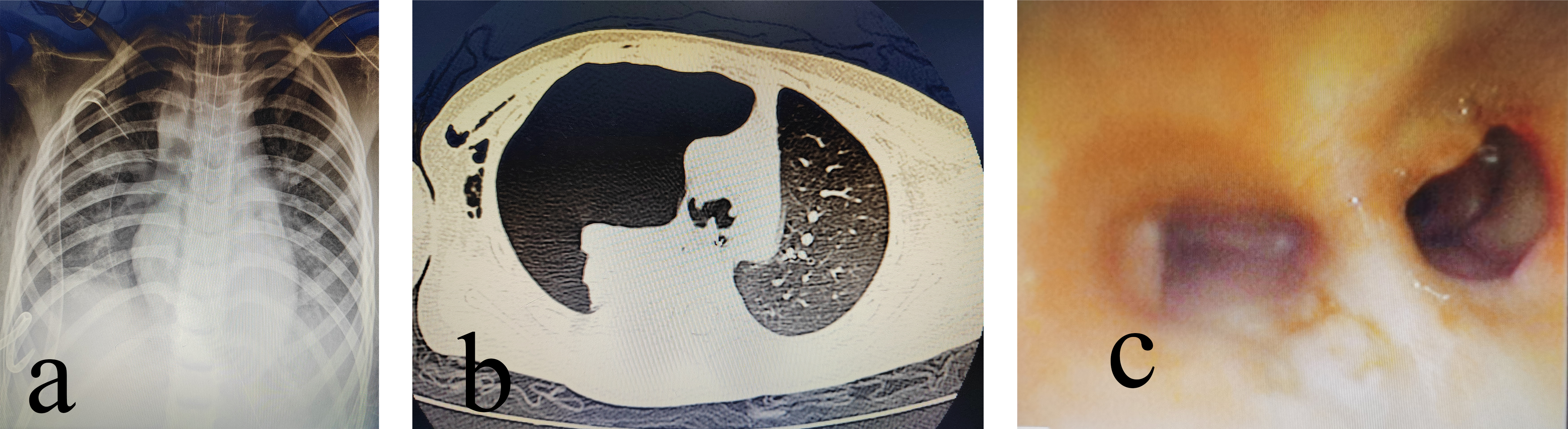

Supplement: Supplementary file 1 — Supplementary Material 1 [file 13019_2024_2828_MOESM1_ESM.tif]

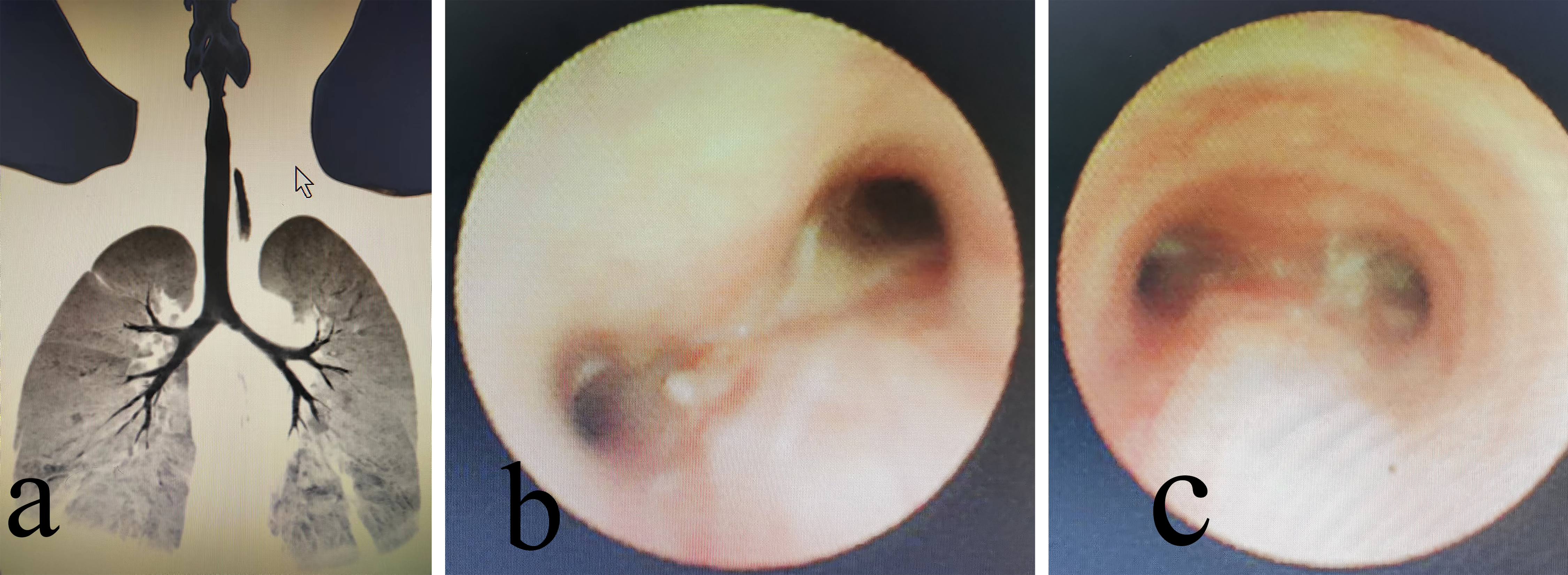

Supplement: Supplementary file 2 — Supplementary Material 2 [file 13019_2024_2828_MOESM2_ESM.tif]

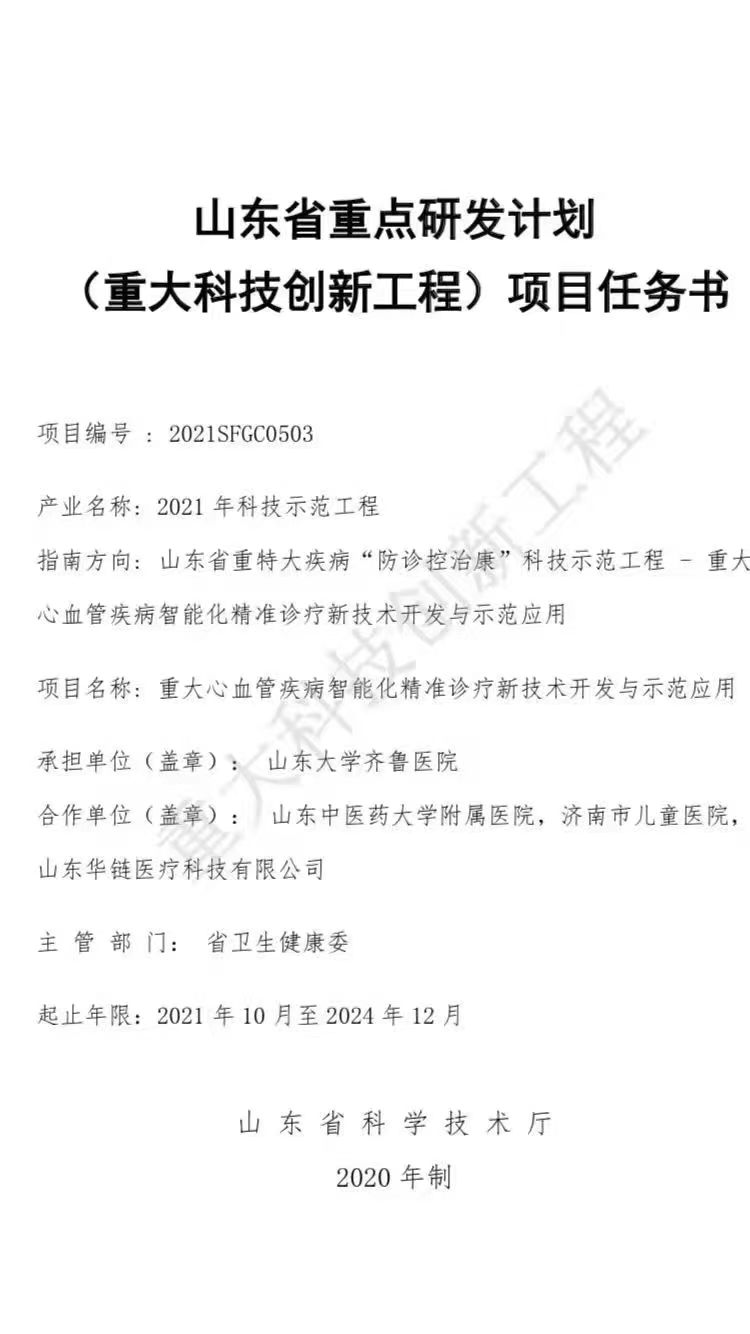

Supplement: Supplementary file 4 — Supplementary Material 4 [file 13019_2024_2828_MOESM4_ESM.jpg]
